# The factors associated with teenage pregnancy among young women aged between 15 and 19 years in Rwanda: a retrospective cross-sectional study on the Rwanda Demographic Health Survey 2019–2020

**DOI:** 10.3389/frph.2024.1453933

**Published:** 2024-12-13

**Authors:** Felix Nduhuye, Emmanuel Kubana, Stella Matutina, David Mwesigye, Athanase Munyaneza, Laetitia Nyirazinyoye

**Affiliations:** ^1^ Department of Epidemiology and Biostatistics, School of Public Health, College of Medicine and Health Sciences, University of Rwanda, Kigali, Rwanda; ^2^ Malaria Division, Rwanda Biomedical Center (RBC), Kigali, Rwanda; ^3^ Department of Radiology and Medical Imaging, Rwanda Military Hospital (RMH), Kigali, Rwanda; ^4^ Einstein Rwanda Research and Capacity Building Program (ER-RCBP), Research for Development (RD Rwanda), Kigali, Rwanda

**Keywords:** teenage, pregnancy, adolescent, Rwanda, Demographic Health Survey

## Abstract

**Background:**

Teenage pregnancy is a significant public health issue and is strongly associated with risky sexual behaviors such as early sexual initiation, unprotected sex, and multiple sexual partners. According to the 2014 World Health Organization report, 11% of all births worldwide were to teenagers aged 15–19 years, with more than 95% of these pregnancies occurring in low- and middle-income countries, particularly in sub-Saharan Africa, which bears much of this burden. In Rwanda, the prevalence of teenage pregnancy has risen from 4.1% in 2005 to 7.3% in 2014, indicating a growing concern. However, there is limited and inconsistent evidence on the factors contributing to teenage pregnancy. Hence, our study aimed to investigate the factors associated with teenage pregnancy. This research seeks to provide valuable insights for targeted interventions, which are urgently needed in light of the increasing rates.

**Methods:**

We employed a cross-sectional study design, utilizing data from the 2019/2020 Rwanda Demographic Health Survey of 3,258 eligible participants aged 15–19 years. To identify factors associated with teenage pregnancy, we performed a bivariate logistic regression analysis. The significant variables from the bivariate analysis were then exported into multivariate logistic regression models, with the results presented as odds ratios (ORs) along with 95% confidence intervals (CIs) and a significance threshold set at 5%.

**Results:**

Our findings indicated that teenagers aged 18–19 years were more likely to experience pregnancy compared to those younger than 17 (OR = 4.2; 95% CI: 2.16–8.37). Adolescents who had engaged in sexual activity 95 times or more had a significantly higher likelihood of becoming pregnant than those with less frequent sexual activity (OR = 13.53; 95% CI: 5.21–35.12). Furthermore, adolescents with parents with a secondary education were 80% less likely to become pregnant compared to those with parents with a primary or no education (OR = 0.2; 95% CI: 0.07–0.63).

**Conclusion:**

Our study revealed that teenage pregnancy is shaped by several individual factors including age and sexual behavior, along with parental education levels. These findings underscore the critical need for targeted sexual education and enhanced family support systems to mitigate teenage pregnancies. Further, longitudinal studies are essential for establishing causality and guiding effective policy development.

## 1 Background

The transition between childhood and adulthood requires particular care and attention from a young person’s caregivers as this stage is accompanied by physiological, psychological, physical, and emotional changes. During this period, some young people are experiencing and engaged in active sexuality and marriage (1, 2). The term “teenage pregnancy” is defined as pregnancy among girls aged 10–19 years old and is associated with neonatal and maternal complications, financial instability, welfare dependency, low level of attended education, abortion, HIV infection, substance abuse, child marriage, and other sexual and reproductive health (SRH) problems. However, this is a public health concern in low- and middle-income countries (LMICs) (3, 4).

The estimated prevalence of pregnancy among adolescent women is 25% worldwide. However, the prevalence of adolescent pregnancy in Africa is recorded at 18.8% with 19.3% in sub-Saharan Africa and 21.5% in Eastern Africa. The prevalence of teenage pregnancy in eastern Africa ranges between 18% and 29% (5). Although the number of teenage pregnancies has fallen considerably in most high-income countries, it remains high in LMICs (6, 7). In 2017, the birth rate among teenagers aged 15–19 years in the United States of America fell significantly to a low teenage fertility rate of 18.8 births per 1,000, a reduction of 7% compared to 2016 (7, 8). In total, 21 million young girls aged 15–19 years old reportedly become pregnant each year and 95% of teenage pregnancies recorded are in LMICs compared to industrialized countries, representing a profound public health concern. For example, childbirth and pregnancy contribute to teenage death with 99% of teenage maternal deaths occurring in LMICs (9, 10).

In LMICs, especially in sub-Saharan African countries, teenage pregnancies have risen sharply (11, 12). In 2013, teenage mothers accounted for more than 50% of all births in this region; the projected number is 101 births for every 1,000 teenagers aged 15–19 years (11, 13). The proportion of young teenage mothers under the age of 18 years has doubled in many African countries, including Mozambique, Niger, Malawi, Uganda, and Cameroon (14, 15).

However, policymakers have developed policies and guidelines to decrease the rate of unintended teenage pregnancy in some LMICs. For instance, Nigeria introduced a curriculum for sex education (10). However, the poor attitude of the teachers and inadequate support from parents and religious leaders has led to a failure to implement this curriculum (7, 8).

Specifically, many policymakers, government officials, religious leaders, and parents fear that talking about sex with young people will only encourage promiscuous behavior. In fact, none of the sex education mandates have made any significant contribution to a decline in teenage pregnancy (16, 17).

In Rwanda, the results of the 2014–2015 Demographic and Health Survey showed an increase in the prevalence of teenage pregnancy. The teenage pregnancy rate rose from 4.1% in 2005 to 7.3% in 2014 (18). In May 2018, the Rwanda Investigation Bureau (RIB) reported 222 cases of rape of adolescent girls under the age of 18 in Rwanda that resulted in pregnancies. The Ministry of Gender and Family Promotion (MIGEPROF) reported that in 2016, 17,000 adolescent girls aged 15–19 years became pregnant (19). Other studies indicate that from 2013 to 2016, 818 teenagers became pregnant in all districts of Rwanda (20). In 2014, the Gender Monitoring Officer of Rwanda (GMO) reported that the issue of teenage pregnancy had reached alarming levels: in 2014 alone, 522 pupils under the age of 18 gave birth and dropped out of school (21, 22). The number of unwanted pregnancies among young girls was alarmingly high in certain districts such as Karongi (Western Province), Gatsibo and Kayonza (Eastern Province), and Gasabo (City of Kigali) (22). Rwandan populations include a large number of adolescents as they represent 50% of the Rwandan population. Teenage pregnancy in Rwanda is not only a health problem, but also a socio-economic and development concern.

Teenage pregnancy is linked to the high risk of sexual and reproductive health problems such as under-age marriage, unwanted pregnancies, a high number of elective abortions performed by non-professionals, an increase in maternal deaths at this age, sexually transmitted infections, HIV and AIDS, and other life-threatening adolescent problems, as well as dropping out of school (23). There is an increased risk of premature delivery, anemia, pre-eclampsia, and low birth weight (14). Many adolescent girls also have limited access to medical services, leading them to resort to unsafe procedures. Approximately 3.9 million adolescents undergo unsafe abortions and approximately 70,000 die as a result (22). Furthermore, they are more likely to develop depression (24). Teenage pregnancy is associated with a higher risk of dropping out of school, which affects their learning, long-term financial security, and future (8, 15). Some research has highlighted a long list of possible risk factors associated with teenage pregnancy, such as low levels of education, lack of negotiation skills, lack of parental supervision, lack of access to contraceptive methods, premature marriage, religion and culture, and the family's economic situation (16, 17). Some strategies that aim to solve these problems involve encouraging sexual abstinence and improving access to contraceptives. Different strategies that combine different methods of support for adolescents need to take female characteristics into account, as well as different cultures, religious beliefs, and population attitudes (18).

Despite the aforementioned increase in teenage pregnancy, there is limited evidence on the factors that contribute to teenage pregnancy. Our study aimed to address this gap by investigating the factors associated with teen pregnancy. By identifying key predictors, the study sought to provide valuable insights for targeted interventions, which are urgently needed in light of the increasing rates. To the best of our knowledge, there is no study on the determinants of teen pregnancy using a recent Rwanda Demographic and Health Survey (RDHS) (19). The results of this study will provide all stakeholders with data for appropriate interventions to reduce teenage pregnancy and the number of early marriages among teenagers. This study will also provide appropriate evidence at the national level to achieve the United Nations Sustainable Development Goal 3 (SDG-3) (7).

## 2 Method and materials

### 2.1 Study design

We conducted a retrospective cross-sectional study using the 2019–2020 RDHS data to analyze factors associated with teenage pregnancy among adolescents aged 15–19 years. This approach allowed us to examine historical patterns and relationships relevant to teenage reproductive health.

### 2.2 Study settings

Rwanda, located in East Africa, is bordered by Uganda to the north, Tanzania to the east, Burundi to the south, and the Democratic Republic of the Congo to the west. With a population of approximately 13.1 million in 2020, Rwanda spans an area of 26,338 km^2^. The country is divided into four provinces and the city of Kigali, encompassing 30 districts, 416 sectors, 2,148 cells, and 14,837 villages. Kinyarwanda, a local language, is the predominant language used alongside English (instructive language) and French. The health system is structured into three tiers: primary healthcare facilities, district hospitals, and specialized referral hospitals. The government has made significant strides in improving access to healthcare services, including SRH services. Healthcare professionals, including doctors, nurses, and midwives, are crucial in providing comprehensive SRH services. They are trained to offer clinical care, counseling, and education, helping to empower adolescents and women to make informed decisions about their health. Further, community health workers (CHWs) also play a critical role in enhancing access to services, particularly in rural areas. They are trained to provide basic healthcare, health education, and referrals, thereby bridging the gap between the health system and underserved populations. CHWs actively promote health initiatives, facilitate access to contraceptives, and provide information about SRH, helping to reduce stigma and increase awareness among adolescents. Despite these efforts, challenges remain in accessing SRH information and services, particularly among adolescents, who face barriers such as stigma, lack of knowledge, and cultural taboos. Although the government has made efforts to improve SRH education and services for young people, many still lack adequate resources and support.

### 2.3 Study population

This research targeted adolescents aged 15–19 years. This selected population encompasses young individuals who participated in the RDHS interviews, representing a crucial demographic for examining teenage pregnancy. The inclusion criteria stipulate that participants must be residents of Rwanda within the specified age range, must have provided informed consent to participate in the survey, and must have complete data on relevant variables related to sexual and reproductive health. Conversely, individuals outside this age bracket, those who did not complete the survey, or who lacked pertinent information regarding teenage pregnancy were excluded from the study. In addition, participants with missing variables essential for analysis were also excluded. This stringent selection process ensures a focused analysis of the sexual and reproductive health challenges faced by Rwandan adolescents, facilitating a comprehensive understanding of the socio-cultural and individual factors that influence teenage pregnancy in the country.

### 2.4 Sample size and sampling techniques

The 2019–2020 RDHS gathered data from 14,634 women aged 15–49 years and 6,513 men aged 15–59 years. A significant portion of the participants was under the age of 30 years, with 53% of women and 55% of men falling within this age group. However, for the purposes of this analysis, only women aged 15–19 years were included, resulting in a study population of 3,258 participants. We were left with this sample after we performed data cleaning and removed participants with missing variables especially those who did not have a teen pregnancy status. The sample for the 2019–2020 RDHS was drawn from a stratified sampling framework established in two stages based on the 2012 census. Stratification involved dividing each district into urban and rural categories, thereby creating a total of 60 distinct sampling strata (25).

### 2.5 Data collection

Data collection was carried out by 17 field teams with 10 supervisors from the National Institute of Statistics of Rwanda (NISR) and the National Reference Laboratory (NRL) who supervised and coordinated all field activities. The fieldwork for the 2019–2020 RDHS was carried out under close supervision starting on 9 November 2019, in the clusters in the 17 districts in the North, West, and East provinces. After completion of the fieldwork in these 17 districts, the teams were dispatched to the final 13 districts. However, due to the COVID-19 pandemic, the fieldwork was suspended from April to June 2020. Data collection resumed on 4 June and was completed on 20 July 2020.

A total of 500 clusters were selected, 112 in urban areas and 388 in rural areas. In total, 26 households were selected from each sample point, for a total sample size of 13,000 households. Because of the approximately equal sample sizes in each district, the sample was not self-weighted at the national level, and weighting factors have been added to the data file so that the results are proportional at the national level. However, all the women and men aged 15–49 years who were either permanent residents of the selected households or visitors who stayed in the household the night before the survey were eligible to be interviewed. Thus, we extracted data for adolescents aged 15–19 years old after obtaining information on teenage pregnancy in all provinces in Rwanda. To access the dataset, the investigator used the DHS program website to request access to the data set and this was approved within 24 h (25).

### 2.6 Materials

A questionnaire and different tools were used in the training. Lectures were held on the technical aspects of biomarker collection, and the other tools used included a video and hands-on demonstrations of the biomarker collection process, instructions on how to fill out the questionnaire and transmittal sheets, and instructions on data quality procedures. In addition, break-out sessions were held daily during which trainees had the opportunity for hands-on practice with both adults and children. A total of three anthropometry standardization exercises were carried out in a community health center. Following the standardization exercises, the results of the exercises were presented. General observations regarding accuracy (difference between the reference value and the participant's value) and precision (difference between the first and second readings) were discussed.

### 2.7 Data analysis

Data analysis was conducted using Stata version 13.0, facilitating both descriptive and analytical assessments. Descriptive statistics included means and standard deviations (SDs) for continuous variables and then frequency and percentages for categorical variables, providing a comprehensive overview of the study participants. For the analytical component, bivariate and multiple logistic regression analyses were employed to identify factors associated with teenage pregnancy. The bivariate analysis helped establish preliminary associations, while multiple logistic regression adjusted for potential confounders, using odds ratios (ORs) to quantify the strength of these associations. Confidence intervals (CIs) at 95% and a statistical significance level set at 5% were utilized to ensure robust findings. This structured approach enabled a thorough examination of the relationships between individual, familial, and contextual factors influencing teenage pregnancy, ultimately contributing to the formulation of targeted interventions and policy recommendations.

## 3 Results

The study sample consisted of 3,258 teenage participants, with ages ranging from 15 to 19 years. The majority of the participants were aged 15–17 years (66.21%). Most of the participants (82.25%) resided in rural areas, while 17.76% lived in urban settings. Geographically, the largest group of participants comes from the Eastern region (30.35%), followed by the Western (21.31%) and Southern (20.92%) regions. Regarding formal education, a little over half of the participants (50.63%) had completed primary education, while 48% had reached secondary school. A significant number of the teenagers (63.82%) were still attending school. Regarding employment, the majority (61.76%) was unemployed. In terms of family background, a large portion of the teenagers (57.95%) had fathers who were still alive. However, media access was limited, with 61.65% of the participants lacking access to radio, and 91.80% having no access to television. Most participants (97.45%) had never been in a union, highlighting their young age and the fact that they were still transitioning into adulthood. Wealth distribution showed a relatively even spread across socio-economic classes, with nearly a quarter (24.99%) falling into the richest category whereas 15.25% of the girls were from the poorest families (Table 1).

**Table 1:** Socio-demographic characteristics of the study participants (*N* = 3,258).

Variable	Frequency	%
Socio-demographic variables		
Age of teenager (years)		
15–17	2,157	66.21
17–19	1,101	33.79
Place of residence		
Urban	579	17.76
Rural	2,680	82.25
Place of residence		
City of Kigali	397	12.18
South	681	20.92
West	694	21.31
North	497	15.25
East	989	30.35
Formal education		
No education	32	0.99
Primary	1,650	50.63
Secondary	1,564	48.00
School attendance		
In school	2,079	63.82
Not in school	87	2.66
Father alive (under 18)		
Yes	1,888	57.95
No	261	8.00
Teen still at school		
Yes	2,079	63.82
No	87	2.66
Employment status of the participants		
Employed	1,246	38.25
Not employed	2,012	61.76
Siblings		
No sibling	3	0.09
Less than 5	97	2.98
5–9	63	1.95
Above 9	6	0.18
Combined wealth index		
Poorest	497	15.25
Poorer	619	19.01
Middle	650	19.95
Richer	678	20.81
Richest	814	24.99
Access to radio		
No	2,008	61.65
Yes	1,250	38.36
Access to TV		
No	2,991	91.80
Yes	267	8.21
Magazine		
No	2,935	90.07
Yes	324	9.93
Marital status		
Never in union	3,175	97.45
Married	1	0.02
Living with partner	73	2.23
Divorced	2	0.07
No longer living together	8	0.23

TV, television.

Our results identified several factors associated with teenage pregnancy: age was a significant factor, with teenagers aged 18–19 years having a much higher likelihood of pregnancy (12.3%) compared to those aged 15–17 years (1.6%), with a highly significant *p* < 0.001.

There was no significant difference between urban and rural areas (*p* = 0.381). However, regional differences were evident, with the East province showing the highest pregnancy rate at 15.4%, though these results were not statistically significant across regions. Education was strongly associated with teenage pregnancy: those with no education had the highest pregnancy rate (25.1%), while those with primary and secondary education had significantly lower rates (7.3% and 2.6%, respectively), with *p* < .001. Employment status was also significant; employed teenagers had a much higher pregnancy rate (8.4%) compared to those who were not employed (3.2%), with *p* < 0.001. Marital status was a critical factor, as teenagers who had ever been in a union were much more likely to be pregnant (19.9%) compared to those who had never been married (3.1%), with a very high significance level (*p* < 0.001). Overall, age, education, employment, and marital status emerged as key factors for teenage pregnancy in Rwanda (Table 2).

**Table 2:** Bivariate analyses of the association between the socio-demographic characteristics and teenage pregnancy in Rwanda.

Variable	Number	Yes			
		%	OR	CI	*p*-value
Age of teenager (years)					
15–17	2,158	1.6	1		
18–19	1,1	12.3	9.98	7.11–14.01	<0.001*
Place of residence					
Urban	579	5	1		
Rural	2,68	5.2	0.86	0.62–1.20	0.381
Province					
City of Kigali	397	0.1	1		
South	681	1	1.07	0.67–1.71	0.777
West	694	3.9	0.77	0.47–1.27	0.308
North	497	8.6	0.87	0.52–1.47	0.622
East	989	15.4	1.16	0.75–1.8	0.502
Education					
No education	32	25.1	1		
Primary	1,65	7.3	0.22	0.11–0.46	<0.001*
Secondary	1,564	2.6	0.08	0.04–0.17	<0.001*
Father alive (for those under 18 years)					
No	87	1	1		
Yes	2,079	1.6	1.53	0.33–7.22	0.586
Don't know	1	0			
Teenage employment status					
Not employed	2,012	3.2	1		
Employed	1,246	8.4	2.53	1.94–3.31	<0.001*
Marital status					
Never married	3,482	3.1	1		
Ever in union	49	19.9	117.93	64.2–216.63	<0.001*

CI, confidence interval.

*Significant of *p*-value.

We found that the teenagers who initiated sexual activity at 18–19 years old were significantly more likely to fall pregnant (42%, *p* = 0.023) compared to those who did so aged 15–17 years. Having multiple sexual partners did not show a significant association, except for those with more than five partners, who had a 100% pregnancy rate. A higher frequency of sexual intercourse was strongly associated with teenage pregnancy, with those having intercourse more frequently (95 times or more) showing a very high likelihood of pregnancy (84.7%, *p* < 0.001). Contraceptive use significantly reduced the risk of pregnancy, with users having a much lower pregnancy rate (69.1%, *p* < 0.001) compared to non-users. Regarding familial backgrounds, education played a key role in reducing teenage pregnancies. Teenagers with a secondary education had the lowest pregnancy rate (2.7%, *p* < 0.001), followed by those with a primary education (7%, *p* = 0.011), while those with no education had a higher pregnancy rate (6.4%). The employment status of the household head was also significant, with unemployed household heads associated with a higher teenage pregnancy rate (8.3%, *p* < 0.001). Household size and wealth quintiles were also influential, with larger households (five to nine members) and lower wealth quintiles (poorest) linked to lower pregnancy rates (*p* = 0.000 and *p* < 0.001, respectively). Concerning access to media and technology, teenagers who read newspapers or magazines at least once a week were significantly less likely to become pregnant (2.7%, *p* = 0.011). Those with less media exposure had higher pregnancy rates, though radio and TV exposure did not show statistically significant differences. For sexual reproductive health characteristics, our findings showed that condom use was strongly protective, with teenagers who used condoms having a significantly lower pregnancy rate (19.4%, *p* < 0.001). Knowledge of family planning methods was also protective, with those knowing about family planning sources for non-users showing a significantly reduced pregnancy rate (0.6%, *p* = 0.003). Domestic violence, whether physical or sexual, did not show a significant association with pregnancy rates. In addition, distance to health facilities was not a significant factor (*p* = 0.448). Overall, age at first sexual intercourse, frequency of intercourse, contraceptive use, education, and employment status of household heads emerged as significant factors associated with teenage pregnancies in Rwanda. Access to media and reproductive health knowledge also played protective roles (Table 3).

**Table 3:** Bivariate logistic regression analyses for behavioral, familial, access to media, and sexual reproductive health factors associated with teen pregnancy.

Variable	Number	Yes			
		%	OR	CI	*p*-value
A. Behavioral factors					
Age at first sexual intercourse (years )					
15–17	3,008	0	1		
18–19	169	42	171.33	89.27–328.8	0.023^a^
Multiple sexual partners					
1	543	31.2	1		
2	116	32.1	1.13	0.66–1.93	0.646
3–5	66	36.7	1.25	0.65–2.45	0.498
Above 5	5	100	1		
Frequency of sexual intercourse					
No sexual intercourse	4,064	1.3	1		
1 time	110	31.8	29.21	15.49–55.10	<0.01^b^
2–4 times	117	38.1	44.77	25.72–77.96	<0.001^c^
5–40 times	55	52.1	73.13	35.88–149.06	<0.001^c^
41–94 times	17	83.9	230.08	56.62–935.04	<0.001^c^
95 times and above	73	84.7	437.30	184.85–1,034.57	<0.001^c^
Use of contraceptive methods					
No	4,27	2.9	1		
Yes	164	69.1	70.846	43.63–115.04	<0.001^c^
B. Familial background factors					
Education level					
No education	392	6.4	1		
Primary	2,321	7	0.30	0.12–0.76	0.011^a^
Secondary	1,696	2.7	0.10	0.04–0.26	<0.001^c^
Gender of HH head					
Male	2,933	5.1	1		
Female	1,503	5.8	1.129	0.80–1.60	0.494
Employment status of HH head					
Employed	1,706	3.5	1		
Not employed	2,729	8.3	2.762	1.98–3.85	<0.001^c^
Number of siblings from biological parent					
No sibling	52	7.3	1		
Less than 5	2,898	4.8	0.58	0.20–1.72	0.325
5–9	1,404	6.1	0.77	0.26–2.36	0.658
Above 9	82	9.5	1.20	0.26–5.55	0.808
HH size					
Less than 5	1,215	10.3	1		
5–9	3,058	3.2	0.267	0.18–0.38	<0.001^c^
Above 9	163	9.2	0.971	0.49–1.91	0.932
HH wealth quintile					
Wealthiest	694	7.8	1		
Fourth	845	5.9	0.82	0.52–1.32	0.423
Middle	911	6.9	0.82	0.52–1.29	0.39
Second	927	3.9	0.49	0.29–0.83	0.008^b^
Poorest	1,058	3.1	0.33	0.18–0.63	0.001^b^
C. Access to media and technology					
Frequency of reading newspaper or magazine					
Not at all	2,779	6.4	1		
Less than once a week	1,133	3.8	0.59	0.38–0.92	0.019^a^
At least once a week	524	2.7	0.42	0.22–0.82	0.011^a^
Frequency of listening to radio					
Not at all	775	6.6	1		
Less than once a week	710	5	0.69	0.40–1.20	0.189
At least once a week	2,95	5.1	0.69	0.47–1.04	0.073
Frequency of watching TV					
Not at all	2,255	5.7	1		
Less than once a week	1,221	4.9	0.846	0.57–1.25	0.4
At least once a week	959	4.9	0.714	0.45–1.14	0.156
D. Sexual reproductive health characteristics					
Domestic violence (teen)					
Physical violence only	101	8.3	1		
Sexual violence only	42	2.2	0.31	0.03–2.94	0.308
Both	32	7.3	0.90	0.16–5.07	0.909
Condom use					
No	255	63.3	1		
Yes	116	19.4	0.12	0.07–0.24	<0.001^c^
Distance to a health facility					
Big problem	833	5.6	1		
Not a big problem	3,603	5.3	0.86	0.58–1.27	0.448
Knows the source of family planning for non-users					
No	3,288	3.5	1		
Yes	983	0.6	0.16	0.05–0.53	0.003^b^

HH, household; TV, television.

^a^ Statistical significance at *p* < 0.05.

^b^ Statistical significance at *p* < 0.01.

^c^ Strongly statistically significant at *p* < 0.001.

The multiple logistic regression analysis identified several significant factors associated with teenage pregnancy. Teenagers who had their first sexual encounter between the ages of 15 and 19 years were at a higher risk of pregnancy (OR = 4.25; 95% CI: 2.16–8.37, *p* < 0.001) compared to those who initiated sexual activity before age 15. Higher frequency of sexual intercourse was also strongly linked to increased odds of pregnancy; for example, those who reported having had sexual intercourse two to four times had an increased likelihood (OR = 2.52; 95% CI: 1.26–5.05, *p* = 0.009), while those having had sexual intercourse 5–40 times and 41–94 times showed ORs of 4.12 (95% CI: 1.74–9.76, *p* = 0.001) and 8.89 (95% CI: 1.91–41.45, *p* = 0.006), respectively. Contraceptive use showed a protective effect, reducing the likelihood of pregnancy (OR = 1.36, 95% CI: 0.66–2.79, *p* < 0.001). Secondary education significantly reduced pregnancy risk, with an OR of 0.21 (95% CI: 0.07–0.63, *p* = 0.005), compared to no education. Employment status was associated with increased odds of teenage pregnancy (OR = 191.66, 95% CI: 1.28–2.87, *p* = 0.002), as was marital status, where married teens had a higher likelihood (OR = 817.37; 95% CI: 4.33–15.44, *p* < 0.001) than their non-married counterparts. Household size also mattered, with larger households (10 or more members) showing a higher risk of pregnancy (OR = 263.52, 95% CI: 1.23–5.63, *p* = 0.013). Access to print media, specifically newspapers or magazines, was associated with lower odds of pregnancy; limited access (less than once a week) had an OR of 0.61 (95% CI: 0.39–0.98, *p* = 0.039), and regular access (at least once a week) had an OR of 0.43 (95% CI: 0.22–0.86, *p* = 0.016). Wealth index and access to radio or TV do not show significant associations, suggesting that socio-economic status and media exposure may have had limited direct influence on teenage pregnancy in this sample (Table 4).

**Table 4:** Multiple logistic regression analysis of the factors associated with teenage pregnancy.

Variable	95%CI				
	OR	Lower bound	Upper bound		*p*-value
Age at first sexual intercourse (years)					
<15	1				
15–19	4.25	2.157	8.370		<0.001^a^
Number of sex partners					
1	1				
2	0.99	0.48	2.00		0.964
3–5	0.74	0.27	1.98		0.546
Frequency of sexual intercourse					
Never	1				
1 time	1.58	0.73	3.44		0.244
2–4 times	2.52	1.26	5.05		0.009^b^
5–40 times	4.12	1.74	9.75		0.001^b^
41–94 times	8.89	190.58	4.15		0.006^b^
95 times and above	1.35	5.21	3.51		<0.001^b^
Use of contraceptive					
No	1				
Yes	1.36	6.65	2.79		<.001^a^
Education					
No education	1				
Primary	0.45	0.16	1.31		0.145
Secondary	0.21	0.07	0.63		0.005^b^
Employment status					
No	1				
Yes	191.66	1.28	2.87		0.002
Marital status					
Never married	1				
Ever married	817.37	4.33	1.54		<0.001^a^
HH size					
0–4					
5–9	0.62	0.38	1.01		0.053
10 or more	263.52	1.23	5.63		0.013^c^
Wealth index					
Poorest	1				
Poorer	112.89	0.60	211.95		0.705
Middle	126.64	0.66	2.42		0.474
Richer	115.58	0.60	2.21		0.661
Richest	0.86	0.41	1.80		0.694
Access to newspapers or magazine					
Not at all	1				
Less than once a week	0.61	0.39	0.98		0.039
At least once a week	0.43	0.22	0.86		0.016^c^
Access to radio					
Not at all	1				
Less than once a week	0.77	0.44	1.34		0.355
At least once a week	0.83	0.54	1.28		0.407
Access to TV					
Not at all	1				
Less than once a week	1.06	0.69	1.62		0.777
At least once a week	0.82	0.49	1.35		0.435

HH, household; TV, television.

^a^ Strongly statistically significant at *p* < 0.001.

^b^ Statistical significance at *p* < 0.01.

^c^ Statistical significance at *p* < 0.05.

## 4 Discussion

The analysis in our study showed that adolescents with parents with secondary education were less exposed to the risk of pregnancy than those with primary education or no education. The results showed a certain similarity in socio-economic factors with other studies carried out in African countries (15). Although some reasonable explanations have pointed to the fact that teenage girls from educated families delay marriage and that lectures on sexual and reproductive health are offered to teenage girls when they talk to their parents, the other explanation for this association is that educated teenage girls are less likely to become pregnant than those with no education. However, another explanation for this association is that teenage girls who become pregnant before they complete secondary education are not in a position to continue it because of the extra care they have to provide for their baby and the responsibilities that come with being a mother. Furthermore, researchers are encouraged to carry out more research into this public health problem (14, 15, 24).

The results showed that adolescent girls whose families were employed were more likely to become pregnant than those whose families were not employed (17). This result had some similarities with studies carried out in some African countries, where socio-economic disadvantage was statistically associated with teenage pregnancy at both the household and community levels. Although being employed or not defined by the level of poverty/income at the household and community levels, in West Africa, poverty at the community level was associated with teenage pregnancy as each time the poverty level increased, the probability of teenage pregnancy increased significantly by 1%. Reasonable factors likely to be associated with teenage pregnancy could be rural or urban environments.

Furthermore, child marriage in poor settings is justified as a means of reducing the economic burden within the household by marrying off female children, thereby reducing household size, and increasing resources through the dowry obtained from the groom at the time of marriage (14, 17).

Our study showed that there was a statistically significant association between a never-married family and teenage pregnancy, with never-married families being more likely to be associated with teenage pregnancy. These results could be attributed to religion. Indeed, in certain contexts, families who do not believe in religion have certain limitations, such as a lack of fear of sin and independence in matters of sexual and reproductive health, which are at the root of these families’ carelessness. Although no research has published these results, researchers are encouraged to carry out such research.

The results of our study show that teenagers in the 15–19 years age group were 4.2 times more likely to become pregnant than those under 15 years. These results are similar to those of studies carried out in developed countries such as America and Australia. Possible attributable factors are genetic and socio-environmental factors. Not surprisingly, delaying sexual debut is one of the aims of many pregnancy prevention efforts, as is (sometimes) improving knowledge, access, and use of contraception (18).

The results of our study showed that adolescents who had sexual intercourse 95 times or more were more likely to become pregnant than those who had sexual intercourse less than 95 times. This may be associated with certain reasonable ideas such as sexual dependence. However, the analysis showed that an increased frequency of sexual intercourse resulted in a statistically significant correlation between the variable and the outcome. Future research on this topic is recommended for policymakers to prevent and monitor teenage pregnancy (17, 18).

## 5 Study strengths and limitations

This study has several limitations that must be acknowledged. First, the retrospective cross-sectional design prevents establishing causal relationships between the identified risk factors and teenage pregnancy. Second, this study relied on a retrospective design with secondary data, which restricts the ability to establish causality and limits the analysis to the variables available in the dataset, potentially missing important contextual factors. For instance, the study did not account for socio-cultural beliefs or perspectives that may play a significant role in influencing teenage pregnancy, which limits a more holistic understanding of the phenomenon. In addition, the exclusion of younger adolescents, specifically those under 15 years old, may have led to either an underestimation or overestimation of the prevalence of teenage pregnancy. This exclusion also restricts the generalizability of the findings to the broader adolescent population aged 10–19 years. Despite these limitations, this study has several notable strengths. First, it utilized a large, nationally representative sample from the RDHS 2019–2020, enabling the results to be generalized to the broader population of young women aged 15–19 years. The use of standardized instruments further strengthens the validity and reliability of the findings. In addition, this study is the first of its kind since 2019 to examine the risk factors associated with teenage pregnancy at the national level in Rwanda, making it a valuable contribution to the existing body of knowledge on adolescent reproductive health in the country. These strengths, combined with the comprehensive nature of the dataset, underscore the importance and relevance of the findings despite the study's inherent limitations.

## 6 Public health implications

The findings of this scholarship have significant public health implications for addressing teenage pregnancy in Rwanda. By identifying key risk factors among young women aged 15–19 years, the study highlights critical areas for intervention, such as improving access to reproductive health education, family planning services, and addressing socio-economic vulnerabilities.

The results underscore the need for targeted public health strategies that can reduce teenage pregnancy rates, particularly in rural and low-income areas. In addition, the study's national scope provides a strong evidence base for policymakers to develop comprehensive programs aimed at empowering adolescents with the knowledge and resources needed to prevent unintended pregnancies, ultimately contributing to better maternal and child health outcomes across the country.

## 7 Future directions

Future research should take a more comprehensive approach to understanding the factors influencing teenage pregnancy by incorporating both qualitative and quantitative methods. A mixed-methods design would allow for a deeper exploration of the socio-cultural, economic, and psychological factors that contribute to teenage pregnancy, providing both statistical rigor and rich, context-specific insights. In addition, longitudinal research is crucial to establish causal relationships and assess the long-term impact of interventions over time, particularly in tracking changes in adolescent behavior, socio-economic conditions, and pregnancy outcomes. Further, incorporating participatory research approaches, such as the Community-Based Participatory Research (CBPR) or Community Development (CD) models, would further enhance the relevance and effectiveness of future interventions on early pregnancies. These approaches engage communities directly in the research process, ensuring that solutions are culturally sensitive, context-specific, and supported by the local population. By involving adolescents, families, and community leaders in the design and implementation of interventions, the CBPR and CD approaches can help foster sustainable behavioral changes and improve the overall health of the community. Furthermore, future studies should consider integrating geospatial analysis to identify geographic areas where teenage pregnancy rates are higher and target interventions accordingly. This could help optimize resource allocation and provide localized public health solutions. Finally, expanding research to include adolescents under the age of 15 years would ensure that interventions target the entire spectrum of at-risk youth, leading to more comprehensive and inclusive strategies for preventing teenage pregnancy.

## 8 Conclusion

The analysis of this study reveals that teenage pregnancy was influenced by a range of individual and family-related factors, particularly among young women aged 15–19 years. Key individual factors, such as age at first sexual encounter, frequency of sexual activity, and contraceptive use, were found to be significantly associated with the likelihood of pregnancy. Furthermore, parental factors such as an increase in parental formal education played a protective role in early pregnancy. These findings emphasize the importance of targeted interventions that address both individual behaviors and family dynamics. Based on our analysis, we recommend that policymakers revise and strengthen existing policies and educational curricula, particularly by focusing on vulnerable categories of youth. There is a need for Behavior Change Communication (BCC) initiatives directed at parents, religious leaders, and educators to foster early sexual health education and encourage preventive behaviors among teenagers. A holistic approach, incorporating both individual and family factors, will be essential for reducing teenage pregnancies and promoting the health and wellbeing of adolescents.

## Data Availability

Publicly available datasets were analyzed in this study. These data can be found here: https://www.dhsprogram.com/data/dataset_admin/login_main.cfm.

## References

[1] Mohr R Carbajal J Sharma B. The influence of educational attainment on teenage pregnancy in low-income countries: a systematic literature review. J Soc Work Glob Community. (2019) 4(1):19–31. 10.5590/JSWGC.2019.04.1.02

[2] Malunga G Sangong S Saah FI Bain LE. Prevalence and factors associated with adolescent pregnancies in Zambia: a systematic review from 2000 to 2022. Arch Public Heal. (2023) 81(1):2–15. 10.1186/s13690-023-01045-y 36805786 PMC9940412

[3] Zemene MA Dagnaw FT Anley DT Dagnew E Zewdie A Haimanot AB Trends and factors associated with teenage pregnancy in Ethiopia: multivariate decomposition analysis. Sci Rep. (2024) 14(1):1–10. 10.1038/s41598-023-50600-8 38278842 PMC10817952

[4] Kassa GM Arowojolu AO Odukogbe ATA Yalew AW. Trends and determinants of teenage childbearing in Ethiopia: evidence from the 2000 to 2016 demographic and health surveys. Ital J Pediatr. (2019) 45(1):1–13. 10.1186/s13052-019-0745-4 31783884 PMC6884780

[5] Worku MG Tessema ZT Teshale AB Tesema GA Yeshaw Y. Prevalence and associated factors of adolescent pregnancy (15–19 years) in east Africa: a multilevel analysis. BMC Pregnancy Childbirth. (2021) 21(1):1–8. 10.1186/s12884-020-03485-8 33771106 PMC7995759

[6] Organization WH. Adolescent pregnancy: issues in adolescent health and development. WHO Publ. *Published Online*. (2014) 36:62–73.

[7] Rulisa S Ntihinyurwa P Ntirushwa D Wong A Olufolabi A. Causes of maternal mortality in Rwanda, 2017–2019. Obstet Gynecol. (2021) 138(4):552–6. 10.1097/AOG.0000000000004534 34623066

[8] Kennedy E Bulu S Harris J Humphreys D Malverus J Gray N. “Be kind to young people so they feel at home”: a qualitative study of adolescents’ and service providers’ perceptions of youth-friendly sexual and reproductive health services in Vanuatu. BMC Heal Serv Res. (2013) 13(1):2–12. 10.1186/1472-6963-13-455 24176059 PMC3842673

[9] Sedgh G Finer L Bankole A. Adolescent pregnancy, birth, and abortion rates across countries: levels and recent trends. J Adolesc Heal. (2015) 56(2):223–30. 10.1016/j.jadohealth.2014.09.007 PMC4852976 25620306

[10] Kumar A Singh T Basu S Pandey S Bhargava V. Outcome of teenage pregnancy. Indian J Pediatr. (2007) 74(10):927–31. 10.1007/s12098-007-0171-2 17978452

[11] Siu WHS Tsai LH Li PR See LC. Prevalence and risk factors for female and male adolescents involved in pregnancy and abortion: a population-based crosssectional study in Taiwan, 2006–2016. J Public Heal. (2023) 31(12):1999–2010. 10.1007/s10389-022-01772-6

[12] Kubana E Munyaneza A Sande S Nduhuye F Karangwa JB Mwesigye D “A comparative analysis of risk factors of malaria “ case study Gisagara and Bugesera District of Rwanda. RDHS 2014/2015. A Retrospective Study. BMC Public Health. (2023) 23:1–9. 10.1186/s12889-023-15104-0 36698124 PMC9875440

[13] Martin J Hamilton B Osterman M Driscoll A. Births: final data for 2018. Natl Vital Stat Reports. (2019) 68(13):1980–2018. 32501202

[14] Hakizimana D Logan J Wong R. Risk factors for pregnancies among females age 15 to 19 in Rwanda: a secondary data analysis of the 2014/2015 Rwanda demographic and health survey (RDHS). J Manag Strateg. (2019) 10(2):49. 10.5430/jms.v10n2p49

[15] Chernick LS Siden JY Bell DL Dayan PS. A qualitative assessment to understand the barriers and enablers affecting contraceptive use among adolescent male emergency department patients. Am J Mens Health. (2019) 13(1):1–11. 10.1177/1557988319825919 30819063 PMC6440070

[16] Christofides N Jewkes R Dunkle K McCarty F Shai NJ Nduna M Risk factors for unplanned and unwanted teenage pregnancies occurring over two years of follow-up among a cohort of young South African women. Glob Health Action. (2014) 7(1):2–10. 10.3402/gha.v7.23719 25150027 PMC4141943

[17] Alum AC Kizza IB Osingada CP Katende G Kaye DK. Factors associated with early resumption of sexual intercourse among postnatal women in Uganda. Reprod Health. (2015) 12(1):1–8. 10.1186/1742-4755-12-1 26585992 PMC4653924

[18] National Institute of Statistics of Rwanda (NISR) [Rwanda], Ministry of Health (MOH) [Rwanda], ICF International. Rwanda Demographic and Health Survey 2014-2015. National Institute of Statistics of Rwanda (2016).

[19] Rugigana E Birungi F Nzayirambaho M. HIV knowledge and risky sexual behavior among men in Rwanda. Pan Afr Med J (2015) 22:380. 10.11604/pamj.2015.22.380.6661 27047620 PMC4796779

[20] Burke H Santo L Bernholc A AAA Chen M. Correlates of rapid repeat pregnancy among adolescents and young women in Uganda. Int Perspect Sex Reprod Heal. (2018) 44(1):8–11. 10.1363/44e5518 29995628

[21] Okoli C Hajizadeh M Rahman M Velayutham E Khanam R. Socioeconomic inequalities in teenage pregnancy in Nigeria: evidence from demographic health survey. BMC Public Health. (2022) 22(1):1–11. 10.1186/s12889-021-12274-7 36096790 PMC9465883

[22] Nuwabaine L QS Kamara K Musaba M. Prevalence and factors associated with teenage pregnancy in Sierra Leone: evidence from a nationally representative demographic and health survey of 2019. BMC Public Health. (2023) 23(1):1–8. 10.1186/s12889-022-14670-z 36941568 PMC10026389

[23] Sengoma J Krantz G Nzayirambaho M Munyanshongore C Edvardsson K Mogren I. Prevalence of pregnancy-related complications and course of labour of surviving women who gave birth in selected health facilities in Rwanda: a health facility-based, cross- sectional study. BMJ Open. (2017) 7(7):2–19. 10.1136/bmjopen-2016-015015 28694344 PMC5734260

[24] Ayele W. Differentials of Early Teenage Pregnancy in Ethiopia, 2000, and 2005. DHS Work Pap No90 (2013). Available online at: http://dhsprogram.com/pubs/pdf/WP90/WP90.pdf

[25] National Institute of Statistics of Rwanda (NISR) [Rwanda], M of H (MOH) [Rwanda] & I. Rwanda Demographic and Health Survey 2019-20. Final Report. Kigali, Rwanda: National Institute of Statistics of Rwanda (NISR) (2021).

