# Supplementary material for: The factors associated with teenage pregnancy among young women aged between 15 and 19 years in Rwanda: a retrospective cross-sectional study on the Rwanda Demographic Health Survey 2019–2020
Source: Front Reprod Health. 2024 Dec 13;6:1453933. doi: 10.3389/frph.2024.1453933 (PMC11671394; doi:10.3389/frph.2024.1453933)
Supplement: Supplementary file 1 [file Datasheet1.pdf]

Table 1. Socio-demographic of study participants.

Description of study participants		
**N=3258**		
**Variables**	**Frequency**	**%**
**Age of teenage women**		
15	810	24.87
16	680	20.88
17	667	20.49
18	504	15.48
19	596	18.30
**Place of residence**		
Urban	579	17.76
Rural	2,680	82.25
**Place of residence**		
City of Kigali	397	12.18
South	681	20.92
West	694	21.31
North	497	15.25
East	989	30.35
**Teenage education level**		
No education	32	0.99
Primary	1,650	50.63
Secondary	1,564	48.00
**School attendance**		
In school	2,079	63.82
Not in school	87	2.66
**Father alive(under18)**		
Yes	1,888	57.95
No	261	8.00
**Father alive(under18)**		
Yes	2,079	63.82
No	87	2.66
**Teenage employment status**		
Employed	1,246	38.25
Not employed	2,012	61.76
**Siblings**		
No sibling	3	0.09
Less than 5	97	2.98
5 to 9	63	1.95
Above9	6	0.18

**wealth index combined**		
Poorest	497	15.25
Poorer	619	19.01
Middle	650	19.95
Richer	678	20.81
Richest	814	24.99
**Radio**		
No	2,008	61.65
Yes	1,250	38.36
**TV**		
No	2,991	91.80
Yes	267	8.21
**Magazine**		
No	2935	90.07
Yes	324	9.93
**Magazine**		

**Marital Status**		
never in union	3,175	97.45
Married	1	0.02
living with partner	73	2.23
Divorced	2	0.07
No longer living together	8	0.23

Table 2: Bivariate analysis of the association between socio-demographic characteristics and teenage pregnancy of young women in Rwanda.

Variables	Teens Women pregnancy			
	RDHS 2019-2020			
		Yes		
	N	(%)	95% CI	P-Value
Study population	3258	5.2		
**Age of teenage**				
15-17*	2,158	1.6		
18-19	1,100	12.3	[7.11456,14.00676]	0
**Place of residence**				
Urban *	579	5		
Rural	2,680	5.2	[0.6181437,1.201677]	0.381
Province				
City of Kigali*	397	0.1		
South	681	1	[0.6702741,1.707466]	0.777
West	694	3.9	[0.4732519,1.266746]	0.308
North	497	8.6	[0.5231021,1.472908]	0.622
East	989	15.4	[0.7502903,1.797644]	0.502
**Teenage education level**				
No education*	32	25.1		
Primary	1,650	7.3	[0.1135399,0.4590541]	0
Secondary	1,564	2.6	[0.0406,0.1736668]	0
**Father alive(for under 18 years)**				
No*	87	1		
Yes	2,079	1.6	[0.327,7.216]	0.586
Don't know	1	0		
**Teenage employment status**				
Not employed*	2,012	3.2		
Employed	1,246	8.4	[1.939,3.312]	0

**Marital status**				
Never married	3,482	3.1		
Ever in union	49	19.9	[64.199,216.633]	0

Table 3: Bivariate analysis of the association with teenage pregnancy of young women with family/ Media factors in Rwanda.

**Variables**	**Categories**	**N**	**Yes (%)**	**95% CI**	**P-Value**
**Bihavioural factors**	**Age at first sexual intercourse**				
	>15 years	3,008	**0**		
	15-19 years	169	42	[89.27,328.8]	**0.023**
	**Multiple sexual partners**				

**Variables**	Categories	N	Yes (%)	95% CI	P-Value
	1 Partner	543	31.2		
	2 Partners	116	32.1	[0.665,1.927]	**0.646**
	3-5 partners	66	36.7	[0.647,2.446]	**0.498**
	Above 5 partners	5	100		
	**Frequency of sexual intercourse**				
	No sexual intercourse	4,064	1.3		
	One time	110	31.8	[15.493,55.101]	**0**
	2-4 times	117	38.1	[25.716,77.955]	**0**
	5-40 times	55	52.1	[35.883,149.062]	**0**
	41 to 94 times	17	83.9	[56.619,935.041]	**0**
	95 times and above	73	84.7	[184.846,1034.573]	**0**

	**Contraceptive use**

	Not using	4,270	2.9		
	Using	164	69.1	[43.627,115.045]	**0**
**Familial background factors**	**Education level**				
	No education	392	6.4		
	Primary	2,321	7	[0.122,0.756]	**0.011**
	Secondary	1,696	2.7	[0.041,0.256]	**0**
	**Gender of HH Head**				
	Male	2,933	5.1		
	Female	1,503	5.8	[0.796,1.602]	**0.494**
	**HH Head employment status**				
	Employed	1,706	3.5		
	not employed	2,729	8.3	[1.978,3.856]	**0**
	**Number of siblings from the teen girl biological mother**				

**Variables**	Categories	N	Yes (%)	95% CI	P-Value
	No sibling	52	7.3		
	Less than 5	2,898	4.8	[0.197,1.717]	**0.325**
	5 to 9	1,404	6.1	[0.257,2.356]	**0.658**
	Above9	82	9.5	[0.263,5.549]	**0.808**
	**HH size**				
	Less than 5	1,215	10.3		
	5 to 9	3,058	3.2	[0.185,0.383]	**0**
	Above9	163	9.2	[0.493,1.912]	**0.932**
	**HH wealth quintile**				
	Wealthiest	694	7.8		
	Fourth	845	5.9	[0.521,1.316]	**0.423**
	Middle	911	6.9	[0.522,1.289]	**0.39**
	Second	927	3.9	[0.294,0.835]	**0.008**
	Poorest	1,058	3.1	[0.177,0.629]	**0.001**

	**Frequency of reading newspaper or magazine**				
	Not at all	2,779	6.4		
	Less than once a week	1,133	3.8	[0.383,0.917]	**0.019**
	At least once a week	524	2.7	[0.215,0.822]	**0.011**
	**Frequency of listening to radio**				
	Not at all	775	6.6		
	Less than once a week	710	5	[0.399,1.199]	**0.189**
	At least once a week	2,950	5.1	[0.468,1.035]	**0.073**
**Access to health information factors**	**Frequency of watching television**				
	Not at all	2,255	5.7		
	Less than once a week	1,221	4.9	[0.572,1.251]	**0.4**
	At least once a week	959	4.9	[0.448,1.137]	**0.156**
	**Domestic violence(Teen)**				

**Variables**	Categories	N	Yes (%)	95% CI	P-Value
	Physical violence only	101	8.3		
	Sexual violence only	42	2.2	[0.035,2.936]	**0.308**
	Both	32	7.3	[0.162,5.0739]	**0.909**
	**Condom use**				
	No	255	63.3		
	Yes	116	19.4	[0.067,0.242]	**0**
	**Distance to the HF**				
	Big problem	833	5.6		
	Not a big problem	3,603	5.3	[0.583,1.269]	**0.448**
	**Knowledge in FP methods**				
**Sexual reproductive factors**	No Method	140	0		
	Know Traditional	4,296	5.5		
	**Knows the source of family planning for non-users**				
	No	3,288	3.5		
	Yes	983	0.6	[0.0487,0.527]	**0.003**

Table 4: Multiple logistic regression analysis of the factors associated with teenage pregnancy in Rwanda; Individual factors

	RDHS-2020		
	**Odds Ratio**	**P-Value**	**95 CI**
**Age at first sex**			
<15			
15-19	4.249842	0	(2.157681 - 8.370636)
**Sex partners**			
1 partner			
2 Partners	0.983675	0.964	(0.4835753 - 2.000964)
3-5 partners	0.738458	0.546	(0.2748089 - 1.984361)
**Sex frequency**			
Never			
One time	1.584239	0.244	(0.7286917 - 3.444272)
2-4 times	2.518562	0.009	(1.256542 - 5.048105)
5-40 times	4.122829	0.001	(1.742307 - 9.755871)
41 to 94 times	8.888389	0.006	(1.90582 - 41.45378)
95 times and above	13.53186	0	(5.214357 - 35.11674)
**Contraceptive use**			
No			
Yes	13.62244	0	(6.654994 - 27.88445)

Table 5. Multiple logistic regression analysis of the factors associated with teenage pregnancy in Rwanda, Family factors.

	RDHS 2020		
	Odds Ratio	P-Value	[95% Conf.
**Education**			
No education			
primary	0.45397	0.145	(0.1569269 - 1.313274)
secondary	0.20955	0.005	(0.0702437 - 0.6251374)
**Employed**			
No			
Yes	1.91659	0.002	(1.280248 - 2.869216)
**Marital status**			
Never married			
Ever married	81.7371	0	(43.27501 - 154.3838)
**HH Size**			
0-4			
5 to 9	0.61882	0.053	(0.3806184 - 1.006091)
10 or more	2.63522	0.013	(1.232578 - 5.634017)
**Wealth**			
Poorest			
poorer	1.12888	0.705	(0.60125 - 2.119547)
middle	1.26641	0.474	(0.6625208 - 2.420762)
richer	1.15577	0.661	(0.6040814 - 2.211282)
richest	0.86282	0.694	(0.413485 - 1.800444)

Table 6: Multiple logistic regression analysis of the factors associated with teenage pregnancy in Rwanda; Exposure to media.

	RDHS 2020		
	Odds Ratio	P value	95% CI
Magazine			
Not at all			
less than once a week	0.613531	0.039	(0.3859444 - 0.9753225)
at least once a week	0.433899	0.016	(0.2197027 - 0.8569243)
**Radio**			
Not at all			
less than once a week	0.768971	0.355	(0.4400395 - 1.343781)
at least once a week	0.833176	0.407	(0.540905 - 1.283372)
**TV**			
Not at all			
less than once a week	1.062357	0.777	(0.6980533 - 1.616784)
at least once a week	0.820786	0.435	(0.4995602 - 1.348565)
