# Supplementary material for: The factors associated with teenage pregnancy among young women aged between 15 and 19 years in Rwanda: a retrospective cross-sectional study on the Rwanda Demographic Health Survey 2019–2020
Source: Front Reprod Health. 2024 Dec 13;6:1453933. doi: 10.3389/frph.2024.1453933 (PMC11671394; doi:10.3389/frph.2024.1453933)
Supplement: Supplementary file 2 [file Datasheet2.pdf]

Table 1: Socio-demographic of study participants (N=3258)

Variables	Frequency	%
**A. Socio- demographic variables**		
**Age of teenage women**		
15-17 years	2157	66.21
17-19 years	1101	33.79
**Place of residence**		
Urban	579	17.76
Rural	2,680	82.25
**Place of residence**		
City of Kigali	397	12.18
South	681	20.92
West	694	21.31
North	497	15.25
East	989	30.35
**Formal education**		
No education	32	0.99
Primary	1,650	50.63
Secondary	1,564	48.00
**School attendance**		
In school	2,079	63.82
Not in school	87	2.66
**Father alive (under18)**		
Yes	1,888	57.95
No	261	8.00
**Teen still at school**		
Yes	2,079	63.82
No	87	2.66
**Employment status for the participants**		
Employed	1,246	38.25
Not employed	2,012	61.76
**Siblings**		
No sibling	3	0.09
Less than 5	97	2.98
5 to 9	63	1.95
Above 9	6	0.18
**Wealth index combined**		
Poorest	497	15.25
Poorer	619	19.01
Middle	650	19.95

Richer	678	20.81
Richest	814	24.99
**Access to radio**		
No	2,008	61.65
Yes	1,250	38.36
**Access to TV**		
No	2,991	91.80
Yes	267	8.21
**Magazine**		
No	2935	90.07
Yes	324	9.93
**Marital Status**		
Never in union	3,175	97.45
Married	1	0.02
Living with partner	73	2.23
Divorced	2	0.07
No longer living together	8	0.23
Notice: TV: Television		

Table 2: Bivariate analysis of the association between socio-demographic characteristics and teenage pregnancy of young women in Rwanda.

Variables	Number	Yes			
		%	OR	CI	p-value
**Age of teenage**					
15-17 years	2,158	1.6	1		
18-19 years	1,1	12.3	9.98	7.11, 14.01	< .001*
**Place of residence**					
Urban	579	5	1		
Rural	2,68	5.2	0.86	0.62, 1.20	0.381
**Province**					
City of Kigali	397	0.1	1		
South	681	1	1.07	0.67, 1.71	0.777
West	694	3.9	0.77	0.47, 1.27	0.308
North	497	8.6	0.87	0.52, 1.47	0.622
East	989	15.4	1.16	0.75, 1.8	0.502
**Education**					
No education	32	25.1	1		
Primary	1,65	7.3	0.22	0.11, 0.46	< .001*
Secondary	1,564	2.6	0.08	0.04, 0.17	< .001*
**Father alive (for under 18 years)**					
No	87	1	1		
Yes	2,079	1.6	1.53	0.33, 7.22	0.586
Don't know	1	0			
**Teenage employment status**					
Not employed	2,012	3.2	1		
Employed	1,246	8.4	2.53	1.94, 3.31	< .001*
**Marital status**					
Never married	3,482	3.1	1		
Ever in union	49	19.9	117.93	64.2, 216.63	< .001*
	CI : Confidence Interval				

Table 3: Bivariate logistic regression analyses for behavioral, family, access to media and sexual reproductive health factors associated with teen pregnancies

Variables	Number	Yes			
		%	OR	CI	p-value
**A. Behavioural factors**					
**Age at first sexual intercourse**					
15-17 years	3,008	0	1		
18-19	169	42	171.33	89.27, 328.8	0.023*
**Multiple sexual partners**					
1 Partner	543	31.2	1		
2 Partners	116	32.1	1.13	0.66, 1.93	0.646
3-5 partners	66	36.7	1.25	0.65, 2.45	0.498
Above 5 partners	5	100	1		
**Frequency of sexual intercourse**					
No sexual intercourse	4,064	1.3	1		
One time	110	31.8	29.21	15.49, 55.10	<.01**
2-4 times	117	38.1	44.77	25.72, 77.96	<.001***
5-40 times	55	52.1	73.13	35.88, 149.06	<.001***
41 to 94 times	17	83.9	230.08	56.62, 935.04	<.001***
95 times and above	73	84.7	437.30	184.85, 1034.57	<.001***
**Use of contraceptive methods**					
No	4,27	2.9	1		
Yes	164	69.1	70.846	43.63, 115.04	<.001***
**B. Familial background factors**					
**Education level**					
No education	392	6.4	1		
Primary	2,321	7	0.30	0.12, 0.76	0.011*
Secondary	1,696	2.7	0.10	0.04, 0.26	<.001***
**Gender of HH head**					
Male	2,933	5.1	1		
Female	1,503	5.8	1.129	0.80, 1.60	0.494
**Employment status for HH head**					
Employed	1,706	3.5	1		
Not employed	2,729	8.3	2.762	1.98, 3.85	<.001***
**Number of sibling from biological parent**					
No sibling	52	7.3	1		
Less than 5	2,898	4.8	0.58	0.20, 1.72	0.325
5 to 9	1,404	6.1	0.77	0.26, 2.36	0.658
Above 9	82	9.5	1.20	0.26, 5.55	0.808
**HH size**					
Less than 5	1,215	10.3	1		
5 to 9	3,058	3.2	0.267	0.18, 0.38	<.001***

Above 9	163	9.2	0.971	0.49, 1.91	0.932
**HH wealth quintile**					
Wealthiest	694	7.8	1		
Fourth	845	5.9	0.82	0.52, 1.32	0.423
Middle	911	6.9	0.82	0.52, 1.29	0.39
Second	927	3.9	0.49	0.29, 0.83	0.008**
Poorest	1,058	3.1	0.33	0.18, 0.63	0.001**
**C. Access to media and technology**					
**Frequency of reading newspaper or magazine**					
Not at all	2,779	6.4	1		
Less than once a week	1,133	3.8	0.59	0.38, 0.92	0.019*
At least once a week	524	2.7	0.42	0.22, 0.82	0.011*
**Frequency of listening to radio**					
Not at all	775	6.6	1		
Less than once a week	710	5	0.69	0.40, 1.20	0.189
At least once a week	2,95	5.1	0.69	0.47, 1.04	0.073
**Frequency of watching TV**					
Not at all	2,255	5.7	1		
Less than once a week	1,221	4.9	0.846	0.57, 1.25	0.4
At least once a week	959	4.9	0.714	0.45, 1.14	0.156
**D. Sexual reproductive health characteristics**					
**Domestic violence (teen)**					
Physical violence only	101	8.3	1		
Sexual violence only	42	2.2	0.31	0.03, 2.94	0.308
Both	32	7.3	0.90	0.16, 5.07	0.909
**Condom use**					
No	255	63.3	1		
Yes	116	19.4	0.12	0.07, 0.24	<.001***
**Distance to health facility**					
Big problem	833	5.6	1		
Not a big problem	3,603	5.3	0.86	0.58,1.27	0.448
**Knows the source of family planning for non-users**					
No	3,288	3.5	1		
Yes	983	0.6	0.16	0.05, 0.53	0.003**
	*Notes: *: statistical significance at p<.05; **: Statistical significance at p<.01; ***: strongly statistical significant at p<.001; HH: Head of Household; TV: Television*				

Table 4: Multiple logistic regression analysis for the factors associated with teenage pregnancy

Variables	95%CI			
	**OR**	**Lower bonds**	**Upper bonds**	***p-value***
**Age at first sex**				
<15	1			
15-19	4.25	2.157.68	8.370	<.001***
**Number of sex partners**				
1 partner	1			
2 Partners	0.99	0.48	2.00	0.964
3-5 partners	0.74	0.27	1.98	0.546
**Frequency of sexual intercourse**				
Never	1			
One time	1.58	0.73	3.44	0.244
2-4 times	2.52	1.26	5.05	0.009**
5-40 times	4.12	1.74	9.75	0.001**
41 to 94 times	8.89	190.58	4.15	0.006**
95 times and above	1.35	5.21	3.51	*<.001***
**Use of contraceptive**				
No	1			
Yes	1.36	6.65	2.79	*<.001****
**Education**				
No education	1			
Primary	0.45	0.16	1.31	0.145
Secondary	0.21	0.07	0.63	0.005**
**Employment status**				
No	1			
Yes	191.66	1.28	2.87	0.002
**Marital status**				
Never married	1			
Ever married	817.37	4.33	1.54	<.001***
**HH Size**				
0-4				
5 to 9	0.62	0.38	1.01	0.053
10 or more	263.52	1.23	5.63	0.013*
**Wealth index**				
Poorest	1			
Poorer	112.89	0.60	211.95	0.705
Middle	126.64	0.66	2.42	0.474
Richer	115.58	0.60	2.21	0.661
Richest	0.86	0.41	1.80	0.694
**Access to the newspapers or magazine**				
Not at all	1			
Less than once a week	0.61	0.39	0.98	0.039

At least once a week	0.43	0.22	0.86	0.016*
**Access to radio**				
Not at all	1			
Less than once a week	0.77	0.44	1.34	0.355
At least once a week	0.83	0.54	1.28	0.407
**Access to TV**				
Not at all	1			
Less than once a week	1.06	0.69	1.62	0.777
At least once a week	0.82	0.49	1.35	0.435

*Notes: *: statistical significance at p<.05; **: Statistical significance at p<.01; ***: strongly statistical significant at p<.001; HH: Head of Household; TV: Television*
